# Inflammasomes: Emerging Central Players in Cancer Immunology and Immunotherapy

**DOI:** 10.3389/fimmu.2018.03028

**Published:** 2018-12-20

**Authors:** Dev Karan

**Affiliations:** Department of Pathology, MCW Cancer Center and Prostate Cancer Center of Excellence, Medical College of Wisconsin, Milwaukee, WI, United States

**Keywords:** inflammation, inflammasome, immunotherapy, cancer, tumor microenvironment

## Abstract

Inflammation has an established role in cancer development and progression and is a key player in regulating the entry and exit of immune cells in the tumor microenvironment, mounting a significant impact on anti-tumor immunity. Recent studies have shed light on the role of inflammasomes in the regulation of inflammation with a focus on the subsequent effects on the immunobiology of tumors. To generate strong anti-tumor immunity, cross-talk between innate, and adaptive immune cells is necessary. Interestingly, inflammasome bridges both arms of the immune system representing a unique opportunity to manipulate the role of inflammation in favor of tumor suppression. In this review, we discuss the impact of inflammasomes on the regulation of the levels of inflammatory cytokines-chemokines and the efficacy of immunotherapy response in cancer treatment.

## Introduction

Tumor development is a complex, multistep process wherein early-stage tumor cells undergo a series of genetic/epigenetic changes and modify the surrounding environment by secreting a plethora of cytokines-chemokines for their advantage. This progressively transformed tumor microenvironment (TME) is infiltrated by different immune cell types that are likely to kill early-stage tumor cells. However, the success or failure of the immune cells to kill tumor cells and to inhibit abnormal cell growth depends on a variety of events occurring at the tumor site. Investigating such interactions both at the cellular and molecular levels in the TME would provide rationale for the development of new treatment approaches.

The role of inflammation is well-accepted in the growth and development of tumors, and it constitutes a leading hallmark in cancer development (1, 2). Inflammation-associated cytokines-chemokines within the TME are secreted by various cell types including tumor and immune cells (residual and infiltrated). Some of the cytokines such as IL-10 and TGF-β suppress the immune cell function and help tumor cells evade the immune system, while cytokines like IL-12, IFN-γ, and TNF-α support immunological functions by enhancing anti-tumor immunity. However, an imbalance in the cytokines-chemokines influx and increased inflammation in the TME results in a tumor-promoting environment. In this review, we provide an update on the potential of targeting inflammasomes to manipulate the role of inflammation in the TME, thereby suppressing tumor promotion activities.

## Inflammasomes and Cancer

Inflammasomes are multi-protein complexes consisting of nucleotide-binding and oligomerization domain (NOD)-like receptor (NLR), the adaptor protein (ASC: apoptosis-associated speck-like protein containing CARD), and procaspase-1. Among NLRs, NLRP1, NLRP2, NLRP3, NLRP4, NLRP6, NLRP12, and NLRP14 are known to form multi-protein complexes and are grouped into a canonical inflammasome pathway (3, 4). Absent in Melanoma 2 (AIM2), also a member of the canonical inflammasome pathway, recognizes aberrant cytoplasmic dsDNA and induces cytokine maturation, release, and pyroptosis (5). The non-canonical inflammasomes include a complex that consists of CARD9, Malt1, Bcl-10, caspase-8, and ASC (6). During non-canonical inflammasome signaling, caspase-1 is known to cleave gasdermin D (GSDMD), resulting in GSDMD-p30 pores in macrophages, releasing IL-1β and IL-18, and inducing pyroptosis. The non-canonical pathway involving NLRP3 inflammasome leading to murine caspase-11 or human caspase-4,-5 maturation is also reported (7). However, later studies confirmed lipopolysaccharides (LPS)-induced toll-like receptor (TLR4)-mediated signaling for activation of caspase-4, -5, and -11 (8–11).

The canonical pathway of inflammasome activation is regulated at both the transcriptional and post-translational levels following two signals (4). The first signal, referred to as the priming signal, is associated with the upregulation of NLRP3 induced by the TLR/nuclear factor (NF)-κB pathway. Alternatively, mitochondrial-derived reactive oxygen species (ROS) can also prime NLRP3 inflammasome activation utilizing TLR4/MyD88 signaling (12). During this priming process, the precursor forms of interleukin (IL) IL-1β, (pro-IL-1β), and IL-18 (pro-IL-18) are also upregulated. The second signal is exerted by various stimuli including pathogen-associated molecular patterns (PAMPs), damage-associated molecular patterns (DAMPs), adenosine triphosphate (ATP), uric acid crystals, or other toxins facilitating the functional assembly of the NLRP3 associated multi-protein complex consisting of ASC and pro-caspase-1 (13, 14). Upon activation, inflammasome assembly regulates the activity of caspase-1, responsible for the proteolytic cleavage of pro-IL-1β and pro-IL-18 to its mature and bioactive forms, hence inducing a variety of biological effects.

Current studies have highlighted the role of inflammasome-mediated inflammation in cancer (2, 15). The most studied and best characterized inflammasome, NLRP3, is an emerging, key player in the development and progression of cancer, and an increased expression of NLRP3 has been associated with multiple cancer types (16–19). Activation of NLRP3 inflammasome has also been shown to promote inflammation-induced tumor growth and metastasis in head and neck cancer and oral squamous carcinoma (20, 21). Contrary to the effects of NLRP3 in the promotion of cancer, studies in colorectal cancer demonstrated that increased NLRP3 inhibits colorectal metastasis. It was demonstrated that IL-18 secretion downstream of NLRP3 inflammasome is associated with increased interferon-gamma (IFN-γ) production and activation of signal transducer and activator of transcription (STAT1) involved in protection against colorectal tumorigenesis (22). Additional mechanisms showed that IL-18 primed natural killer (NK) cells trigger FasL-induced apoptosis in the tumor (23). In a pre-clinical model of colitis-associated cancer (CAC), mice lacking the inflammasome adaptor proteins PYCARD (ASC) and caspase-1 demonstrated increased disease outcome, morbidity, and polyp formation. Expression of inflammasome component NLRP3 was also negatively associated with the progression of hepatocellular carcinoma (24). Immunohistochemical analysis in prostate biopsies showed almost a uniform expression of NLRP3 and did not reveal any association with prostate cancer progression (15).

Besides NLRP3, studies describing the role of other inflammasome components are limited. Melanoma studies demonstrated the role of NLRP1 in tumor promotion by increasing inflammasome activity and suppressing apoptosis in metastatic melanoma (25). Due to limited studies in the literature, the role of NLRC4 seems inconsistent. One study demonstrated a negative correlation between NLRC4 and colitis-associated tumors. Utilizing azoxymethane (AOM)/dextran sodium sulfate (DSS) model, it was shown that NLRC4 knockout mice developed increased tumor volume (26). However, in another study, yet with the same model system, no difference in tumor growth was found between NLRC4-deficient and wild-type mice (26, 27). Similarly, the absence of NLRP6 accelerated colitis-associated tumors in mice, while its presence was demonstrated to suppress inflammation and carcinogenesis. It was found that NLRP6 helps to preserve the integrity of epithelial barriers and hence prevents adenoma formation (28). Immunohistochemical analysis of archival prostate biopsy specimens showed a significant increase in NLRP12 expression in prostate cancer tissues as compared to that in benign tissues (15). On the contrary, it was demonstrated that NLRP12 suppresses colon inflammation and tumorigenesis through negative regulation of NF-κB signaling (29, 30). Increased activation of AIM2 inflammasome is associated with the early course of acute pancreatitis (31). AIM2 inflammasome is also reported to play a critical role in the development of human prostatic diseases (32). AIM2, an interferon (IFN) inducible protein, is constitutively down-regulated in prostate cancer, however, IFN-induced AIM2 inflammasome activation leads to increased production of IL-1β and IL-18 in prostate cancer cell lines.

Emerging studies on the expression of inflammasome components ASC and caspase-1 also support the critical role of these molecules in tumor growth and development. In many cancer types, including prostate, breast, lung, and glioblastoma, ASC is downregulated due to hypermethylation (33–36). ASC contributes to the process of apoptosis and functions as a Bax adaptor by translocating Bax to mitochondria (37). In fact, ASC is essential in bridging the activity of NLRP inflammasomes and pro-caspase-1. ASC exerts its functional activity by translocating from the nucleus to the cytoplasm and localizes with the inflammasome components NLRP and pro-caspase-1 (38). Taken together, the multi-protein complex of inflammasomes drives a cascade of pro-inflammatory cytokines regulating various cellular activities.

IL-1 is the most extensively characterized inflammasome-related cytokine promoting cancer, whereas IL-1β is a pleiotropic inflammatory cytokine associated with cell proliferation, differentiation, tissue regeneration, and immune cell regulation. Additionally, the role of IL-1β in angiogenesis, tumor promotion, metastasis, resistance to chemotherapy, and immunosuppression are well-described (39, 40). Several other members of the IL-1β family, including IL-18 and IL-33, are processed and activated by caspase-1, a driving component of inflammasome activity (41). Upregulation of IL-33 has been reported in tumor growth and metastasis in lung, colorectal, and gastric cancer (42). An increased serum level of IL-33 is associated with poor prognosis in breast and lung cancer (43–45). Thus, the inflammasome-mediated production of pro-inflammatory cytokines has been identified as a critical modulator of disease outcome. Multiple studies have explored the role of inflammasomes in carcinogenesis and anti-tumor activities with conflicting observations (46–50). Pro-tumor and anti-tumor role of NLRP inflammasome is summarized in Table 1. We observed that in a majority of tumor types, increased level of NLRP contributes to tumor progression while in colitis-associated tumors, NLRP showed anti-tumor activity. Such a dissociated role for the NLRP inflammasome in colitis-associated tumors could be attributed to microbiota, which orchestrate the colonic microenvironment in association with inflammasomes. However, further studies are needed to determine their tissue-specific functional activation of inflammasome components.

**Table 1:** Pro-tumor and anti-tumor activity of inflammasomes in various cancer types.

**Inflammasome complex proteins**	**Pro-tumor activity with increased expression in cancer**	**Anti-tumor activity with increased expression in cancer**
NLRP1	Melanoma, Breast cancer	Colorectal cancer
NLRP3	Breast cancer, Lung cancer, Gastric cancer, Melanoma, HNCC, OSCC, GBM, and colitis-associated cancer	Colorectal cancer, HCC
NLRC4	Gastric cancer, Breast cancer	Either anti-tumor or no effect in colitis-associated cancer
NLRP6	Breast cancer, Gastric cancer	Colitis-associated cancer
NLRP12	Prostate cancer, Gastric cancer	Colon cancer
AIM2	Acute pancreatitis, Breast cancer; Lung cancer; Prostatic diseases	Not known

*HNCC, Head and neck cell cancer; OSCC, Oral squamous cell carcinoma; HCC, Hepatocellular carcinoma; GBM, Glioblastoma*.

## Inflammasomes in Myeloid Cells

Initially, NLRs were thought to be expressed in innate immune cells of monocyte lineage. However, NLRP3 is central to inflammasome research and is expressed in multiple cell types, including monocytes, macrophages, granulocytes, dendritic cells (DCs), epithelial cells, and osteoblasts (51–54). NLRP3 inflammasome is activated by a number of DAMPs and PAMPs recognized by TLRs, which signal via myeloid differentiation marker (MyD88) and Toll-IL-1 receptor (TIR) domain-containing adaptor-inducing interferons (TRIF), and pro-inflammatory cytokines that direct the adaptive immune response (55). Inflammasomes are also an integral part of adaptive Th1 cell response, where assembly of NLRP3 inflammasome is shown in CD4^+^ T cells leading to caspase-1 dependent IL-1β secretion (56). In fact, DCs naturally express TLRs, a family of pattern recognition receptors (PRR), which recognizes PAMPs and DAMPs on the cell surface, whereas NLRs serve as cytosolic sensors (57). In the event of danger signals, NLR and TLR synergize to expand the maturation of DCs, migration, antigen presenting function, and adaptive immune system activation. However, in the absence of TLR involvement, inflammasome activation stimulates an immunosuppressive behavior in DCs (57).

The information on the molecular profiling of other inflammasome components is sporadic. NLRP6 is expressed in myeloid cells such as granulocytes, dendritic cells and macrophages and is a potential regulator of innate immunity, wherein lack of NLRP6 protects mice against bacterial pathogens, which is attributed to an increased number of monocytes and neutrophils (58, 59). In response to microbial infection, assembly of NLRP7 has been shown in human macrophages (60, 61). Lack of NLRP10 has been associated with impairment in DC functions and initiation of adaptive immunity (62, 63). Bacterial pathogens also activate AIM2 inflammasome in macrophages and DCs, resulting in caspase-1 activation and inducing pyroptotic cell death to control bacterial infection (64, 65). These studies highlight the emerging importance of various inflammasomes in the cells of myeloid origin which are helpful in maintaining homeostasis and regulating inflammation, infection, and immunity.

## Emerging Concept of Inflammasomes in Cancer Immunotherapy

Cancer immunotherapy has evolved considerably from approaches such as cell-specific targeting, TME manipulation, systemic inhibition of immune suppressor cells to immune check-point inhibitors, and their subsequent combinations. However, T cells and natural killer (NK) cells remain the primary source of ammunition for targeting cancer. T cells are viewed as most suitable for antigen-specific tumor targeting while NK cells kill both tumor cells and virally infected cells. In the development of effector T cells and NK cells, antigen presenting cells (APCs) remain central in augmenting strong anti-tumor response. To generate antigen-specific effector T cell response, APCs such as dendritic cells (DCs) present the antigen to the cytotoxic T cells (CTL), known as signal-I, which is accompanied by signal-II associated with co-stimulatory molecules. Combination of these two signals leads to fully activated T cells with anti-tumor potential. However, an additional signal-III is also necessary for the proliferation and differentiation of effector cells with enhanced anti-tumor immunity (66). DCs also perform a critical task with regard to translating the interplay between innate and adaptive immunity, and these interactions are being utilized to improve current anti-cancer immunotherapies. While previous studies were more focused on T cell-based immune targeted therapies, the potential of NK cell-based therapies are well-recognized and are advancing the field of immunotherapy in conjunction with T cells. Recent studies showed that NK cells are also regulatory cells engaged in reciprocal interactions with dendritic cells, macrophages, T cells, and endothelial cells (67, 68). There is a strong triangular relationship, directly or indirectly between APCs, NK cells, and T cells. In fact, an optimum modulation of APCs (e.g., DCs) is critical to generate highly tumor-reactive NK or T cells.

A divergent process of immune cell (including DCs) mobilization, which is secretion of various cytokines, plays a significant role in the generation of anti-tumor immunity. Cytokines and chemokines expressed by DCs have a significant impact on the development as well as recruitment and priming of T helper cells. Additionally, the presence of IFN-α activates NK cells and the Th1 cytokines such as IFN-γ and IL-12, which helps in augmenting the antigen-specific immunity emphasizing the critical role of cytokines in immune cell regulations and tumor immunity. IFN-γ supports anti-tumor activities in multiple ways by upregulating major histocompatibility complex-I (MHC-I) in tumor cells, inhibiting the process of angiogenesis and tumor cell proliferation, and at the same time augmenting cytotoxic effector cell functions of CTLs, NK cells, macrophages, and CD4^+^ T cells polarization (69). On the contrary, IFN-γ has also been associated with tumor promotion by supporting the resistance of tumor cells to immune cell-mediated killing (70). Similarly, TNF-α mediates anti-tumor immunity through simultaneous recruitment and activation of macrophages and DCs. However, dysregulated TNF-α signaling is also associated with the promotion of tumor cell growth via the mediation of epithelial-mesenchymal transition (EMT) (71). Thus, the regulation of pro-inflammatory cytokines following inflammasome activation will have a potential impact on immune cell interactions and the process of differentiation.

The central role of IL-33, a member of the IL-1 family, in inducing tumor-promoting type 2 responses has recently gained attention. Treatment of IL-33 in tumor-bearing mice impairs the functional activity of NK cells and dendritic cells and influences macrophages to M2 polarization, thus, suppressing innate and adaptive anti-tumor immunity (42). Administration of IL-33 in a murine model of breast cancer resulted in increased tumor growth and development of metastases, which correlated with increased intra-tumoral numbers of IL-13-producing innate lymphoid cells (ILCs), IL-13 receptor 1-expressing myeloid-derived suppressor cells (MDSCs), and regulatory T cells (T-regs) (72). IL-13 has been shown to activate tumor-promoting MDSCs and their production of anti-inflammatory transforming growth factor-beta (TGF-β) (73). In addition, IL-13 can polarize macrophages toward a pro-tumorigenic M2 phenotype and actively participate in immune evasion (74). Therefore, a tight regulation of cytokines-chemokines will help in the maintenance of homeostatic balance and is potentially regulated via inflammasomes.

Since inflammasomes influence the production of cytokines and immune cells differentiation by regulating the functions of APCs, it seems obvious that the role of inflammasomes could directly be associated with events at the site of the tumor. The role of immune suppressor cells such as T-regs, MDSCs, and tumor-associated macrophages (TAMs) is well-established in the promotion of tumor growth and metastasis. These cells suppress the immune effector cell functions in multiple ways and hamper the clinical impact of immunotherapy. Therefore, chemotherapeutic drugs are used for selective killing of immune suppressor cells to enhance the clinical impact of immunotherapy approaches. Further substantiating the role of inflammasomes in the immunobiology of cancer is the significantly higher activation of NLRP3 inflammasome observed during chemotherapy treatment. It is suggested that dying tumor cells release ATP that is sensed by the P2X7 receptor of DCs leading to NLRP3 activation and is associated with chemo-resistant tumor growth. Additionally, chemotherapy triggers cathepsin B release in myeloid-derived suppressor cells, activating the NLRP3 inflammasome leading to MDSC-derived IL-1β and angiogenesis and promoting tumor growth and metastasis (75, 76). IL-1β is also known to enhance the production of IL-17 by CD4^+^ T cells, which in turn favors angiogenesis and tumor growth (77).

It is interesting to note that the inflammasome component NLRP3 also impairs the impact of anti-tumor vaccine. Dendritic cell-based vaccination of NLRP3-deficient mice bearing melanoma tumors showed a significant increase in survival as compared to their respective controls. This improved survival was attributed to low numbers of MDSCs in NLRP3-deficient tumors suggesting the role of NLRP3 in facilitating the migration of MDSCs to the TME (78). In a model of chemically-induced carcinogenesis, mice lacking NLRP3 showed low tumor burden and suppression of metastasis (79). NLRP3-deficiency induces NK cell infiltration and is associated with increased production of chemokines CCL5 and CXCL9 promoting the anti-metastatic activity of NK cells. Similarly, in a breast cancer model, deficiency of inflammasome components (NLRP3 or caspase-1 knockout) reduced tumor growth and metastasis and was correlated with reduced infiltration of MDSCs within the tumors (80). In a mouse model of pancreatic ductal adenocarcinoma (PDA), it was observed that NLRP3 promotes the expansion of immune-suppressive macrophages. NLRP3 signaling in macrophages drives the differentiation of CD4^+^ T cells into tumor-promoting Th2, Th17, and T-regulatory cell types, while suppressing Th1 cell polarization and cytotoxic CD8^+^ T cell activation. Inflammasome signaling also modulates IL-12 secretion, hence affecting T helper cell polarization. Subsequent inhibition of NLRP3 signaling or mice deficient in inflammasome components (NLRP3, ASC, or caspase-1) showed immunogenic reprogramming of innate and adaptive immunity within the TME and were protected against PDA (81). These preclinical studies provide a proof-of-concept that targeting the inflammasome reduces the quality and quantity of immune suppressor cells and hence inhibits tumor growth and metastasis. Therefore, targeted inhibition of inflammasome activation will help to generate a balancing act of pro-inflammatory cytokine-chemokine in the TME and is likely to restore the immune surveillance, augmenting anti-tumor immunity.

## Inflammasome Inhibitors and Therapeutic Intervention

So far, we described that the absence of inflammasomes helped in protecting mice against tumor growth and was associated with reduced immune suppressor cells in the circulation as well as in the tumor microenvironment. However, the use of synthetic compounds or small molecule inhibitors for *in vivo* targeting of NLRP3 and examining the infiltrating lymphocytes in the tumor bed are just at the initial stage. Several inhibitors have been proposed to target NLRP3 inflammasome activation *in vitro* and *in vivo*, resulting in reduced levels of IL-1β, and inflammasome components associated with anti-inflammatory diseases (82, 83). Using *in vitro* studies in cell lines, we also showed that a dietary agent withaferin-A disintegrates the inflammasome complex and modulates multiple cytokines and chemokines associated with inflammation and cancer (13). Interestingly, intraperitoneal administration of MCC950 to block NLRP3 inflammasome activity significantly reduces the number of T-regs, MDSCs, and TAMs, and increases the numbers of CD4^+^ and CD8^+^ T cells in mice. MCC950 inhibited NLRP3 activation and caspase-1 dependent IL-1β processing, and hence improved anti-tumor immune response in head and neck squamous cell carcinoma (84). Another small molecule inhibitor, andrographolide, protected mice against colitis-associated cancer (CAC) through mitophagy-mediated NLRP3 inhibition (85). Andrographolide was administered intragastrically daily following AOM/DSS-induced CAC in C57BL/6 mice and significantly attenuated colitis progression and tumor burden. Although regulation of immune cells was not determined, the protection of mice against CAC was associated with the disruption of inflammasome assembly and reduced IL-1β secretion. This is in contrast with the CAC studies in NRLP3- or caspase-1-deficient mice, which are susceptible to aggressive AOM/DSS-induced CAC. Since inflammasomes are involved in host defense mechanism against infection and autoinflammatory diseases, a complete knockout of inflammasome components (e.g., NLRP3, ASC, or caspase-1) may cause an imbalance in intestinal microbiota and/or cytokines-chemokines profile promoting an aggressive colitis progression. This clearly indicates that the inhibition of inflammasome activity using small molecules has preventive and therapeutic potential in reducing pro-inflammatory events at the tumor site and providing an opportunity to boost the efficacy of immunological manipulations in cancer treatment.

## Conclusions

**Figure 1:**
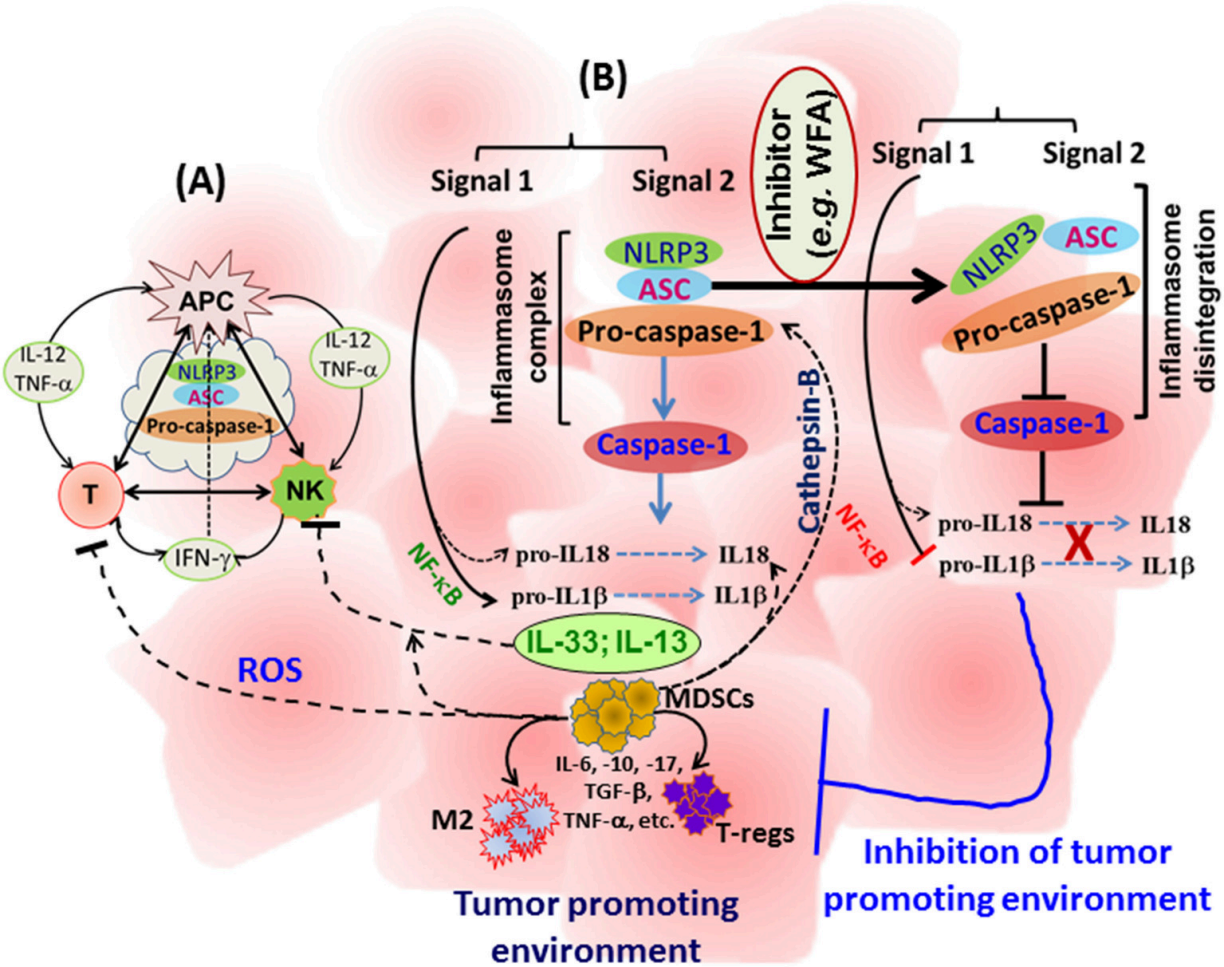
A schematic representation of the cross-talk between APC, NK, and T cells with a potential influence of inflammasome regulating immune cell activity **(A)**, and a summary of events involved in tumor growth and metastasis and a proposed mechanism to block inflammasome activity **(B)**. IL-1β and IL-18 are well-known primary targets; however, multiple cytokines-chemokines could be simultaneously reduced in the tumor microenvironment with subsequent inhibition of immune suppressor cells, providing a space to enhance anti-tumor immunity. WFA: Withaferin-A.

Manipulation of the body's immune system has emerged as one of the most promising approaches leading the way for successful immunotherapies targeting various cancers. In addition to classical approaches of vaccination-induced anti-tumor immunity, development of programmed T cells, transfer of apoptosis-resistant T cells, and metabolically more active or cancer-specific chimeric antigen-receptor (CAR) T cells are being tested in clinics. However, the cytokines-chemokines profile is critical in maintaining the anti-tumor immunity as well as the proliferation and interaction of immune cells. Multiple studies have analyzed the impact of NLRP3 inflammasomes in auto-immune and auto-inflammatory diseases using NLRP3 gene-specific knockout mice. However, studies are emerging that aim to determine the role of NLRP3 in the regulation of immune cells in a tumor setting. As described above, inflammasome components regulate the cytokines-chemokines profile, bridge the innate, and adaptive immune responses, and hence, impact immune cell functions. While inflammasome components revealed an anti-tumor effect in colon and CAC, it is evident that in most of the solid tumors, the NLRP inflammasome is associated with tumor promotion and metastasis (Table 1). It is likely that small molecule based targeting of specific inflammasome components (NLRP1, NLRP3, NLRC4, NLRP6, NLRP12, or caspase-1), rather than using gene-specific knockout strains, might divulge useful information about the role of inflammasomes in mice protected against colon and CAC and hence strengthening the role of inflammasomes in tumorigenesis. Therefore, targeted inhibition of inflammasomes might provide a novel opportunity to manipulate the immunobiology of cancer to augment the efficacy of immunotherapeutic approaches. A schematic representation of inflammasome-centralized cross-talk between APC, NK, and T cells and the potential events in cytokine-chemokine modulations following inhibition of NLRP3 inflammasome components, is summarized in Figure 1.

## Author Contributions

DK conceived the concept, searched the literature and wrote the manuscript.

### Conflict of Interest Statement

The author declares that the research was conducted in the absence of any commercial or financial relationships that could be construed as a potential conflict of interest.
